# Electrical detection of *ortho*–*para* conversion in fullerene-encapsulated water

**DOI:** 10.1038/ncomms9112

**Published:** 2015-08-24

**Authors:** Benno Meier, Salvatore Mamone, Maria Concistrè, Javier Alonso-Valdesueiro, Andrea Krachmalnicoff, Richard J. Whitby, Malcolm H. Levitt

**Affiliations:** 1 School of Chemistry, University of Southampton, Southampton SO17 1BJ, UK

## Abstract

Water exists in two spin isomers, *ortho* and *para*, that have different nuclear spin states. In bulk water, rapid proton exchange and hindered molecular rotation obscure the direct observation of two spin isomers. The supramolecular endofullerene H_2_O@C_60_ provides freely rotating, isolated water molecules even at cryogenic temperatures. Here we show that the bulk dielectric constant of this substance depends on the *ortho*/*para* ratio, and changes slowly in time after a sudden temperature jump, due to nuclear spin conversion. The attribution of the effect to *ortho*–*para* conversion is validated by comparison with nuclear magnetic resonance and quantum theory. The change in dielectric constant is consistent with an electric dipole moment of 0.51±0.05 Debye for an encapsulated water molecule, indicating the partial shielding of the water dipole by the encapsulating cage. The dependence of bulk dielectric constant on nuclear spin isomer composition appears to be a previously unreported physical phenomenon.

## Abstract (web summary)

Water molecules can exist in one of two nuclear spin states—*ortho* or *para*. Here, the authors look at the bulk dielectric constant of water molecules enclosed in fullerenes (capable of rotating even at cryogenic temperatures) and show that it changes as the water molecules convert from *ortho* to *para*.

## Graphical abstract

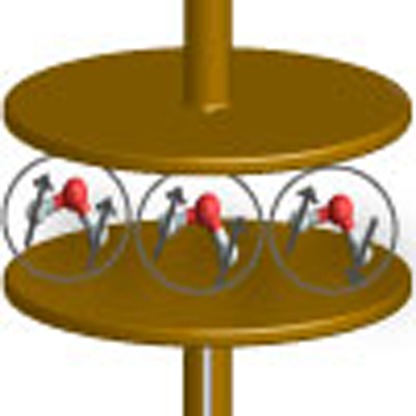

Water, like dihydrogen, has two different spin isomers, called *ortho* and *para*, which have different spin state symmetries. In *ortho*-water, the spin state of the two proton nuclei is symmetric under particle exchange and the total nuclear spin has quantum number *I*=1. In *para*-water, the spin state is antisymmetric and has nuclear spin quantum number *I*=0. Water spin isomerism is of relevance to a broad range of scientific fields from nuclear magnetic resonance (NMR) to astrophysics^1^,^2^,^3^,^4^,^5^, and closely related to long-lived nuclear spin states, which also involve the slow interconversion of nuclear singlet and triplet states^6^,^7^.

Physical properties of dihydrogen H_2_, such as heat capacity or thermal conductivity, depend on the concentration of *ortho* and *para* spin isomers^8^. Do the spin isomers of water also have different bulk properties? Since water, unlike dihydrogen, possesses an electric dipole moment, the spin isomers of *ortho* and *para* water are expected to display a distinct response to electric fields. This effect was predicted theoretically^9^; and observed in beam experiments^10^, but no bulk properties have been reported.

Although it is feasible to separate *ortho-* and *para*-water molecules in rarified molecular beams^5^,^10^, it remains challenging to study the separated isomers in the condensed phase, since rapid proton exchange obscures the spin isomerism in bulk water, and strong intermolecular interactions usually quench the molecular rotation at low temperatures. Spin isomer-enriched water may be captured in an inert gas matrix and studied using infrared spectroscopy^11^,^12^, but this approach provides little control over the molecular environment.

In contrast, the supramolecular endofullerene H_2_O@C_60_, composed of C_60_ carbon cages that each encloses a single water molecule, forms a well-defined lattice. The synthesis of this material provides macroscopic quantities of a stable substance that contains isolated and freely rotating water molecules^13^,^14^. It has been studied under a very wide range of physical conditions using various spectroscopic techniques^1^,^2^,^5^. Dielectric measurements were made on a single crystal of H_2_O@C_60_, but without anticipating, or observing, a dependence on spin isomer composition^15^.

Figure 1a,b shows the molecular structure of H_2_O@C_60_ and the four lowest rotational energy levels as determined by neutron scattering, neglecting the observed splitting of the *ortho*-water ground state^1^,^3^. The energy levels are similar to those of water in the gas phase indicating that the water rotation is unhindered even at cryogenic temperatures. The thermal equilibrium fraction of *ortho*-water molecules as a function of temperature is shown in Fig. 1c, using the energy levels of Fig. 1b and taking into account the degeneracies of the rotational levels^2^,^16^. The equilibrium fraction changes rapidly in the vicinity of 15 K. *Ortho–para* conversion may therefore be induced by (i) allowing the sample to reach complete equilibrium at a temperature >15 K, (ii) rapidly cooling to <15 K and (iii) studying the behaviour of the sample as a function of time at the constant low temperature.

Here we demonstrate that the bulk dielectric constant of H_2_O@C_60_ depends on the spin isomer composition of the encapsulated water molecules. We find a time-dependent change in dielectric constant at 5 K that is due to different molecular polarizabilities of the *ortho* and *para* ground states. The polarizabilities are extracted from the capacitance data and compared with a theoretical prediction that only requires knowledge of the dipole moment of H_2_O@C_60_ and the rotational constants of water. The dipole moment is estimated from a high-temperature measurement of the molecular polarizability and found to be in very good agreement with recent predictions of 0.5±0.1 Debye^17^,^18^,^19^.

## Results

### Dielectric constant

**Figure 2:**
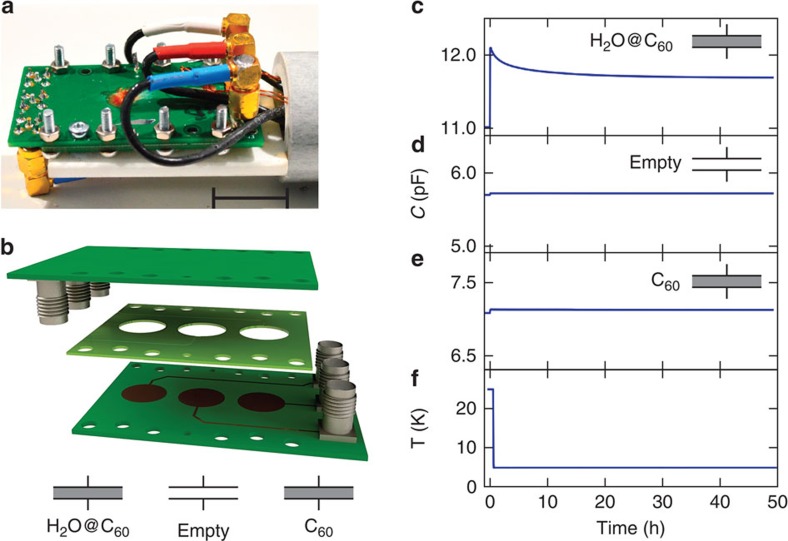
Detection of *ortho–para* conversion in H_2_O@C_60_. (**a**) Photograph and (**b**) exploded view of the sample cell consisting of two printed circuit boards (PCBs, green) and a 210 μm thick G10 spacer used to centre the sample pellets between the electrodes of the two PCBs, forming three independent capacitor cells. Scale bar length in **a** is 2 cm. Following a temperature jump from 25 to 5 K at the time origin, the capacitance of the three cells is measured as a function of time. (**c**) The capacitance of the cell loaded with H_2_O@C_60_ decreases monotonically due to *ortho–para* conversion. The capacitances of (**d**) the empty cell and (**e**) the cell loaded with C_60_ change slightly as the temperature is changed, but are constant after the temperature jump. The temperature measurement is shown in **f**.

The dielectric response of water on *ortho*–*para* conversion is demonstrated with the apparatus shown in Fig. 2 (see Supplementary Methods for details). Three capacitors are measured simultaneously: one is filled with a 4:1 homogeneous mixture of H_2_O@C_60_ and C_60_, one is filled with pure C_60_, and one is left empty. The apparatus was used to measure (1) the variation of dielectric constant with time at low temperature, which is related to *ortho–para* conversion and (2) the variation of dielectric constant with temperature above 50 K. The low-temperature experimental data are shown in Fig. 2c–f. Following equilibration at 25 K, the temperature is rapidly decreased to 5 K and stabilized within ±30 mK for the remaining time of the experiment. The capacitance of the H_2_O@C_60_ cell decreases over approximately 50 h. No significant variation is observed for the capacitor filled with pure C_60_, or the empty capacitor. All measurements are performed at constant frequency and temperature, so that *ortho*–*para* conversion is the only process that can account for the decay shown in Fig. 2c. Other possible influences such as geometric drift or gradual penetration of liquid helium into the cell may be discounted since the pure C_60_ and empty capacitors do not show similar behaviour.

### Nuclear magnetic resonance

**Figure 3:**
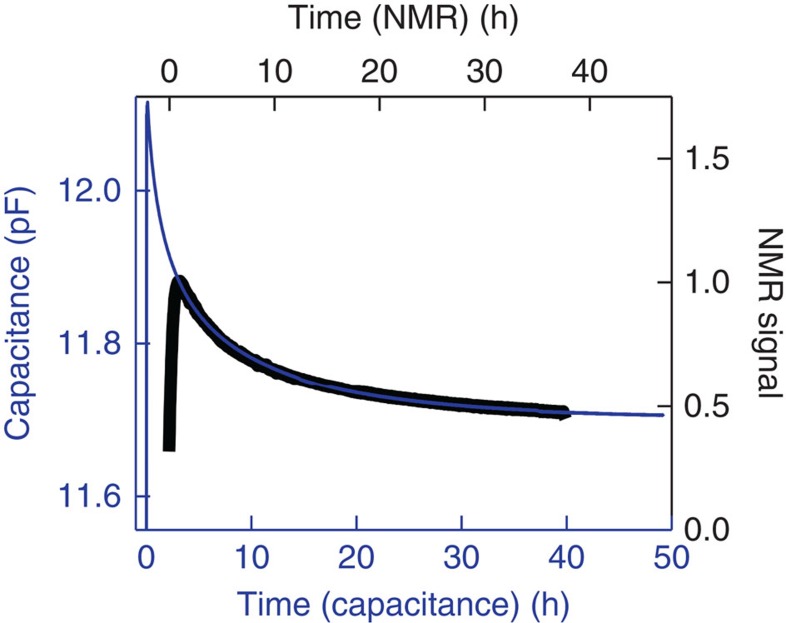
Comparison with NMR data. Capacitance (blue) and ^1^H NMR signal (black) as a function of time, measured on the same sample batch. The origins of the capacitance and NMR time axes are defined by the temperature jump.

Proton NMR measurements provide an independent measure of nuclear spin conversion in H_2_O@C_60_ since the ^1^H NMR signal is proportional to the *ortho* spin isomer fraction Φ. At a temperature of 5 K, the spin isomer conversion takes many hours and follows second-order kinetics^2^. The 600 MHz proton NMR signal amplitude after rapid sample cooling from 25 to 5 K was measured in a magnetic field of 14.1 T using a separate apparatus but the same sample batch as the one used for the capacitance measurements. The NMR signal is shown in Fig. 3 (black points). The NMR results show a small but significant *ortho* fraction persisting even after 40 h at 5 K. The reasons for the metastable *ortho* fraction in this sample batch are currently unknown.

The results of NMR and capacitance measurements are compared in Fig. 3. As described in the Supplementary Discussion, the NMR data were shifted in time by 2.2 h with respect to the capacitance data to take into account the smaller *ortho* fraction at the time the final temperature is reached in the NMR experiment, compared to the capacitance measurement. This difference in initial *ortho* fraction is due to the slower cooling rate of the NMR probe compared to the capacitance probe, which leads to an initial depletion of the *ortho* fraction through the enhanced *ortho*–*para* conversion rate at high temperature^2^,^20^. The similar behaviour of the capacitance and NMR data strongly supports the conclusion that the decay in dielectric constant of H_2_O@C_60_ is due to nuclear spin isomer conversion (see Supplementary Discussion).

The NMR signal intensity and the dielectric constant both display a rapid initial increase on sample cooling. However these rapid initial changes have a different origin in the two measurements. In NMR, *ortho*-water gives rise to a signal and *para*-water does not. The NMR signal of *ortho*-water depends on the polarization of the Zeeman substates of each *ortho* level. The Zeeman polarization, and hence the NMR signal, follow the Curie law in this temperature regime and increase on decreasing temperature. As detailed in the Supplementary Discussion and Supplementary Fig. 4, the dielectric constant also follows a Curie law at higher temperatures, but strong deviations are observed at low temperatures where only a few rotational states are populated.

## Discussion

The dielectric constants of the H_2_O@C_60_/C_60_ sample and the pure C_60_ sample may be calculated from the known geometry of the capacitors. From the dielectric constant one can estimate the microscopic molecular polarizability of H_2_O@C_60_. We denote the molecular polarizabilities of C_60_ filled with H_2_O by *α*_·_ and of empty C_60_ by *α*_○_, and the corresponding volume densities by *N*_·_ and *N*_○_, respectively. The molecular polarizabilities are linked to the dielectric constant by the Clausius-Mossotti relation^21^:


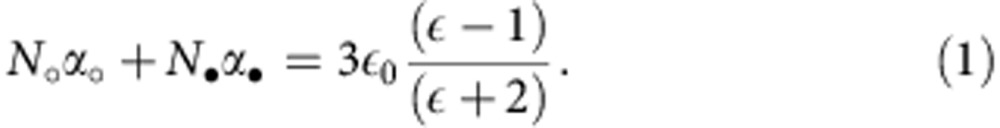


Knowledge of the empty cage polarizability *α*_○_ and the empty and filled cage volume densities *N*_○_ and *N*_·_ enables one to compute *α*_·_, the molecular polarizability of H_2_O@C_60_. To study the effect of the encapsulation of water in C_60_, we measured the capacitance of the three cells as a function of temperature between 50 and 250 K, and used the Clausius-Mossotti relationship to estimate the molecular polarizabilities for both C_60_ and H_2_O@C_60_. These data are interpreted in terms of a temperature-independent deformation polarizability, plus a temperature-dependent orientational polarizability associated with partial molecular alignment along an applied electric field^22^. In agreement with previous reports^23^ we find a deformation polarizability volume for empty C_60_ of 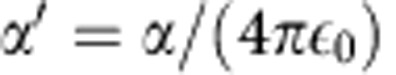 of 87±5 Å^3^ and additionally a weak thermally activated polarizability volume of 5±1 Å^3^ at low temperature^24^ (see Supplementary Fig. 1 and Supplementary Discussion). For H_2_O@C_60_, the molecular polarizability may be written as *α*_·_=*α*_○_+*α*_·_^orient^+Δ*α*_∞_, where *α*_·_^orient^ denotes the orientational polarizability due to the water dipole moment and Δ*α*_∞_ corresponds to a change in deformation polarizability. The observed change in deformation polarizability is Δ*α*_∞_=17±3 Å^3^; significantly larger than a recent theoretical prediction^18^. The reasons for the discrepancy are currently unknown. The orientational polarizability *α*_·_^orient^, shown in Supplementary Fig. 2, is found to follow a Debye relationship corresponding to a dipole moment of *μ*=0.51±0.05 Debye.

The enhanced deformation polarizability of H_2_O@C_60_ may be used to calculate its orientational polarizability volume at low temperatures (Supplementary Fig. 3). As detailed in the Supplementary Discussion, the time-dependent capacitance and NMR data may be combined to estimate the individual polarizability volumes of the *ortho* and *para* ground states. We obtain *α*^*ortho*^′=43±5 Å^3^ and *α*^*para*^′=29±3 Å^3^. These results are in acceptable agreement with the theoretical estimates of 32±6 Å^3^ and 23±5 Å^3^ for the *ortho* and *para* polarizability volumes of the free rotor states obtained by quantum theory^16^,^25^, as implemented in the CMIStark software package^26^, taking into account the reduced dipole moment of 0.51±0.05 Debye (see Supplementary Fig. 4, Supplementary Table 1 and Supplementary Discussion). The relationship between the orientational polarizability and the free rotor wavefunctions is discussed in ref. 26.

The observed change in the bulk dielectric constant, as encapsulated water converts from the *ortho* to the *para* spin isomer, is due to a change in molecular polarizability on spin conversion, with conversion kinetics in agreement with previous NMR studies. Separate molecular polarizabilities for the *ortho* and *para* ground states are extracted from the experimental data. The dipole moment of H_2_O@C_60_ has been measured experimentally and is found to be in good agreement with recent computational predictions^17^,^18^,^19^. A calculation of the *ortho* and *para* ground state polarizabilities based on this dipole moment yields good agreement with the capacitance data. The different response of *ortho* and *para* water to electric fields provides a sensitive alternative means to study the spin isomers. The phenomenon opens up the prospect of using Kelvin probe force microscopy^27^ to study water spin isomers on a single-molecule level.

## Additional information

**How to cite this article:** Meier, B. *et al.* Electrical detection of *ortho–para* conversion in fullerene-encapsulated water. *Nat. Commun.* 6:8112 doi: 10.1038/ncomms9112 (2015).

## Supplementary Material

Supplementary Information Supplementary Figures 1-4, Supplementary Table 1, Supplementary Discussion, Supplementary Methods and Supplementary References

## Figures and Tables

**Figure 1:**
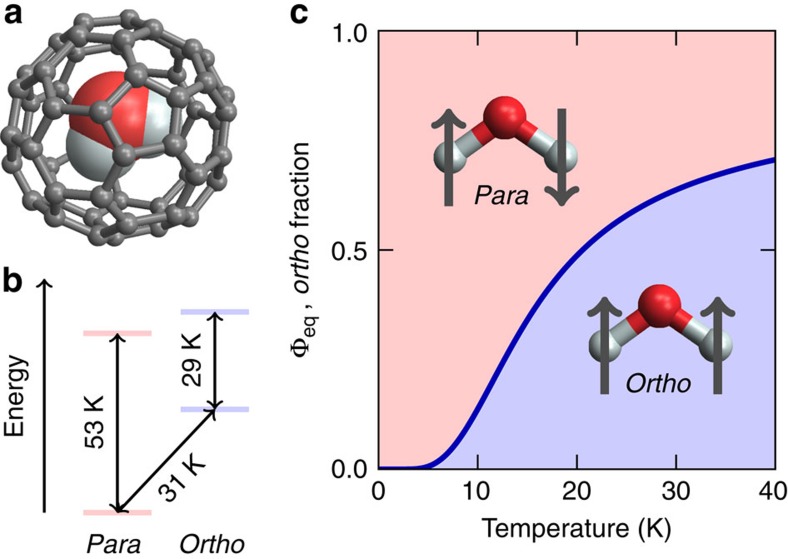
*Ortho* and *para* water inside C_60_. (**a**) Molecular structure of the endofullerene H_2_O@C_60_ and (**b**) the lowest rotational energy levels, in temperature units^1^,^3^. The small splitting of the *ortho*-H_2_O ground state is ignored for simplicity. (**c**) Blue line: fraction of *ortho*-water Φ _eq_ as a function of temperature in thermal equilibrium.
