# Supplementary material for: Electrical detection of ortho–para conversion in fullerene-encapsulated water
Source: Nat Commun. 2015 Aug 24;6:8112. doi: 10.1038/ncomms9112 (PMC4560827; doi:10.1038/ncomms9112)
Supplement: Supplementary Information — Supplementary Figures 1-4, Supplementary Table 1, Supplementary Discussion, Supplementary Methods and Supplementary References [file ncomms9112-s1.pdf]

Supplementary Figures

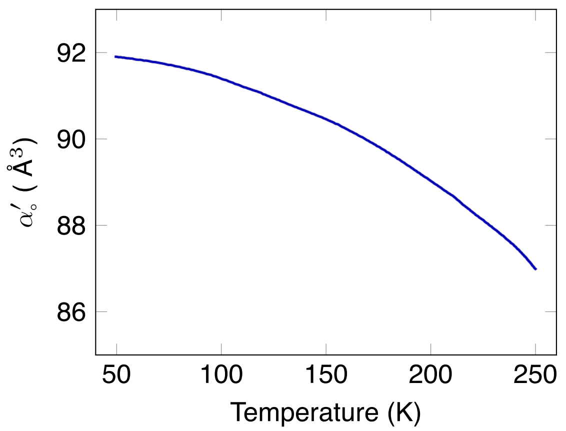

Supplementary Figure 1. Polarizability volume of C_{60} versus temperature, dervied from the capacitance of the capacitor filled with empty C_{60}.

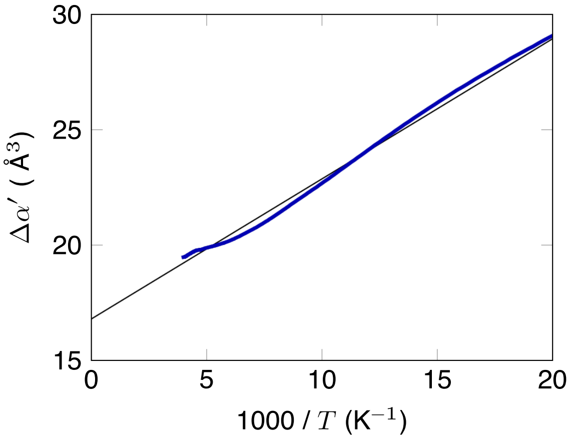

Supplementary Figure 2. Difference between polarizability volumes of $H_2O@C_{60}$ and C_{60} versus inverse temperature for $T \geq$50 K (blue curve). The black line shows a linear regression to a Debye law, $\Delta\alpha(1/T) = \Delta\alpha_\infty + \mu^2/(3kT)$, yielding a dipole moment of 0.51 Debye.

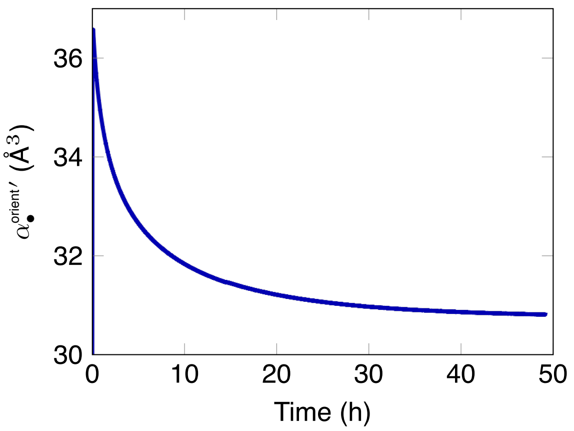

Supplementary Figure 3. Orientational polarizabilitiy volume $\alpha_{\bullet}^{\mathrm{orient}\prime}$ of $H_2O@C_{60}$ at 5 K, after a temperature jump from 25 K. The decay is due to ortho-para conversion.

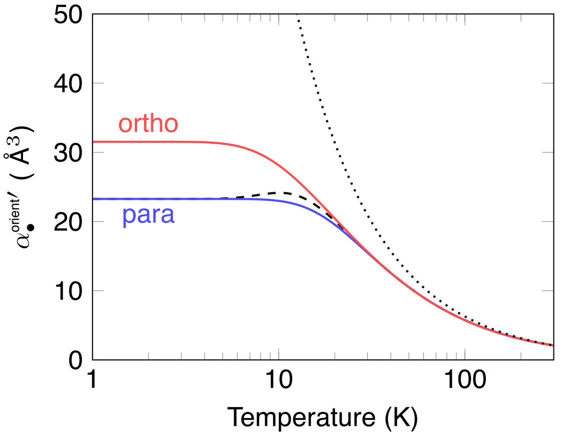

Supplementary Figure 4. Calculated orientational polarizability volume of $H_2O@C_{60}$ versus temperature, assuming a dipole moment of 0.51 Debye and gas phase rotational constants. The solid red and blue curves show seperate Boltzmann averages over the ortho and para rotational states up to $J = 7$, respectively. The dashed black curve shows the full thermal equilibrium polarizability volume obtained by averaging over both ortho and para states and taking into account the three-fold degeneracy of the ortho states. The dotted line corresponds to the Curie law.

Supplementary Tables

$\vert JK_aK_CM\rangle$	Energy (K)	α' (Å^3)
$\vert 0000\rangle$	0	23.3
$\vert 1010\rangle$	34.8	9.2
$\vert 101\pm1\rangle$	34.8	42.7
$\vert 1110\rangle$	54.0	-11.2
$\vert 111\pm1\rangle$	54.0	9.1

Supplementary Table 1. Energies and polarizability volumes α' for the first three rotational levels of $H_2O@C_{60}$, assuming a dipole moment of 0.51 Debye and gas phase rotational constants.

Supplementary Discussion

NMR

The presence of a significant NMR signal at long times indicates a significant ortho fraction persisting at 5 K, far greater than the fraction expected in full thermal equilibrium. This long-term ortho fraction, denoted $\Phi_{5\mathrm{K}}(\infty)$, was estimated as follows: Both the NMR data and the capacitance data were fitted to a second order model of the form $f(t) = B + A/(1 + kt)$. For a second order model, the rate constant k depends on the time origin of the fit; a shift in time $t' = t + \Delta t$ leads to a change in rate constant $k' = k/(1 + kt)$. The NMR data was hence shifted in time to match the capacitance data rate constant, for which the temperature jump was nearly instantaneous. This time shift takes into account the slower cooling rate of the NMR probe, which leads to a reduced ortho fraction after the temperature jump, compared to the capacitance measurement: Cooling of the NMR and capacitance probes took 62 min and 8 min, respectively.By extrapolating the NMR fit to $t' = 0$ the initial NMR signal for an instantaneous temperature jump may be estimated to be 24 % larger than the maximum NMR signal observed during the experiment. Assuming full equilibration at 25 K, the ortho fraction for an instantaneous jump is $\Phi_{5\mathrm{K}}(0) = \Phi^{\mathrm{eq}}_{25\mathrm{K}}$. Both NMR[3] and IR[12] data indicate fast nuclear spin conversion at temperatures above 15 K, so the assumption of thermal equilibrium at 25 K is expected to be valid.

Since the NMR signal is proportional to the ortho-fraction, the ratio between final and initial NMR intensities equals the ratio between final and initial ortho-fractions:

$$\frac{B^{\mathrm{NMR}}}{1.24} = \frac{\Phi_{5\mathrm{K}}(\infty)}{\Phi^{\mathrm{eq}}_{25\mathrm{K}}}, \tag{1}$$

where B^{NMR} denotes the final NMR intensity. Based on this equation we estimate a final ortho-fraction of $\Phi_{5\mathrm{K}}(\infty) = 0.13$ for both NMR and capacitance data.

Capacitance and Ortho-Fraction

At low temperatures only the ortho and para ground states are populated and the $H_2O@C_{60}$ orientational polarizability can be written as

$$\alpha^{\mathrm{orient}}_{\bullet} = \Phi\alpha^{\mathrm{ortho}} + (1 - \Phi)\alpha^{\mathrm{para}} = \Phi(\alpha^{\mathrm{ortho}} - \alpha^{\mathrm{para}}) + \alpha^{\mathrm{para}}. \tag{2}$$

Insertion into the Clausius-Mossotti equation (Eq. (1) in the main text) yields:

$$N_\circ \alpha_\circ + N_\bullet \left(\alpha_\circ + \Delta\alpha_\infty + \Phi(\alpha^{\mathrm{ortho}} - \alpha^{\mathrm{para}}) + \alpha^{\mathrm{para}}\right) = 3\epsilon_0 \frac{(\epsilon - 1)}{(\epsilon + 2)}, \tag{3}$$

that is, the left hand side of the Clausius-Mossotti equation depends linearly on the ortho-fraction Φ.

The dielectric constant $\epsilon = C/C_0$ is determined as the ratio of the measured capacitance C (Fig. 2c in the main text) and the calculated capacitance of an empty capacitor with the same geometry, $C_0 = \epsilon_0 \pi r^2/d$, with $r = 6.5$ mm and $d = 300$ µm, cf. section .

At 5 Kelvin ϵ varies in the range (2.3, 2.4) and we can expand the right-hand side of the above equation to first order in ϵ at $\bar{\epsilon}$ =2.35 to yield

$$\Delta\Phi \approx 3\epsilon_0 \frac{3}{(\bar{\epsilon} + 2)^2} \frac{\Delta\epsilon}{\alpha^{\mathrm{ortho}} - \alpha^{\mathrm{para}}}, \tag{4}$$

where $\Delta\epsilon = \epsilon - \bar{\epsilon}$. The first-order expansion is accurate to within 0.05% for experimentally encountered values of ϵ, hence the capacitance depends linearly on the ortho-fraction.

Polarizability of C_{60} and $H_2O@C_{60}$

The polarizability of C_{60} is readily calculated from the dielectric constant of the pure C_{60} sample ($N_\bullet = 0$) and plotted as polarizability volume $\alpha'_\circ = \alpha_\circ/(4\pi\epsilon_0)$ in Fig. S1. The temperature was changed at a rate of approximately 2 K / min. In agreement with previous reports[4] we find a deformation polarizability volume of approximately 87±4 Å^3. We also observe an additional weak thermally activated polarizability at low temperatures reminiscent of that reported for a C_{60} single crystal by Alers et al.[5]. The exact temperature-dependence of the dielectric constant of C_{60} remains controversial with Yan et al.[6] reporting an increase in capacitance upon *increasing* the temperature.

Figure S2 shows the difference in polarizability volumes, $\Delta\alpha' = \alpha'_\bullet - \alpha'_\circ$, versus $1/T$ for temperatures T above 50 K. In this regime, the difference in polarizability between full and empty cages is found to follow a Debye relationship, $\Delta\alpha = \Delta\alpha_\infty + \alpha_\bullet^{\mathrm{orient}}(T)$ where α_∞ is termed the deformation polarizability and $\alpha_\bullet^{\mathrm{orient}}(T) = \mu^2/(3kT)$ is referred to as orientational polarizability[7]. A linear regression yields $\Delta\alpha'_\infty$ =17±3Å^3 and $\mu = 0.51\pm0.05$ Debye where the error estimates include instrumental factors. These quantities have the following significance: (1) the positive value of $\Delta\alpha$ indicates that the presence of the water

molecule in the cage increases the deformation polarizability by 20%. (2) The estimated dipole moment of 0.51 D for $H_2O@C_{60}$, when compared with the dipole moment of 1.85 D for a free water molecule[8], indicates that the H_2O molecule induces an opposite electric dipole in the carbon cage; the two dipoles substantially cancel each other. The estimate of 0.51±0.05 D for the overall dipole moment of $H_2O@C_{60}$ is in good agreement with recent density functional calculations[9].

Orientational Polarizability of $H_2O@C_{60}$ at 5K

The orientational polarizability of $H_2O@C_{60}$, following a temperature jump from 25 to 5 K, is shown in Fig. S3. It may be written as $\alpha^{\mathrm{orient}}_{\bullet,\,5\mathrm{K}}(t) = \Delta\alpha(t) - \Delta\alpha_\infty$, where $\Delta\alpha(t)$ is derived from the low-temperature measurement and $\Delta\alpha_\infty$ is derived from Fig. S3. Assuming that the equilibrium ortho-fraction at 25 K, $\Phi^{\mathrm{eq}}_{25\mathrm{K}}$ is essentially preserved during the temperature jump, the orientational part of the polarizability is initially given as $\alpha^{\mathrm{orient}}_{\bullet,\,5\mathrm{K}}(0) = \Phi^{\mathrm{eq}}_{25\mathrm{K}}\alpha^{\mathrm{ortho}} + (1 - \Phi^{\mathrm{eq}}_{25\mathrm{K}})\alpha^{\mathrm{para}}$ where α^{ortho} and α^{para} denote the polarizabilities of the ortho and para ground states. The final ortho-fraction, $\Phi_{5\mathrm{K}}(\infty) = 0.13$, is estimated from the NMR measurements as discussed above and corresponds to the final polarizability, $\alpha^{\mathrm{orient}}_{\bullet,\,5\mathrm{K}}(\infty) = \Phi_{5\mathrm{K}}\alpha^{\mathrm{ortho}} + (1-\Phi_{5\mathrm{K}})\alpha^{\mathrm{para}}$. Solving these two equations yields the polarizabilities of the ortho and para ground states. The uncertainties of α^{ortho} and α^{para} are estimated as ± 5 Å^3 and ± 3 Å^3 by error propagation of uncertainties of 0.05 and 0.02 in the initial and final ortho fraction and 2 Å^3 in the initial and final orientational polarizability.

Quantum Mechanical Calculation of Ortho and Para Polarizabilities

The rotational states of water are classified by the asymmetric rotor quantum numbers J, K_a and K_c where J is the total angular momentum and K_a and K_c are its projections along the axis with the smallest and the largest moments of inertia in the molecular fixed frame, respectively. The projection of the electric dipole moment along the applied electric field is characterized by a fourth quantum number $M = -J, \ldots, J$. The interaction of the electric dipole moment of water with an applied electric field leads to a small energy shift, known as the Stark effect. The shift depends on the rotational state and can be calculated via numeric diagonalization of the Hamiltonian expressed in symmetric rotor wavefunctions[10];

the method is implemented and detailed in the CMIStark software package[11]. For all fields of practical concern a constant orientational polarizability α is obtained as second derivative of the energy E with respect to the field ε, $\alpha = -\partial^2 E/\partial\varepsilon^2$. In the linear regime the calculated orientational polarizabilities depend only on the rotational constants of water and scale quadratically with its dipole moment μ. The results for the first three rotational states, along with their energies are given in Tab. S1 as polarizability volumes. Assuming gas-phase rotational constants[13] and a dipole moment of μ = 0.51±0.05 Debye, one can calculate the polarizability volumes of the para and ortho rotational ground states as 23 ± 5 Å^3 and 32±6 Å^3, where the latter is an average over M = -1,0,1. The ratio of these values, 0.74, is independent of the dipole moment of water.

Infrared and inelastic neutron scattering data indicate a good correspondence between the energy levels of $H_2O@C_{60}$ and free water[12], except for an unexplained splitting in the ortho-H_2O ground state, and a possible shift in the rotational energies relative to the gas phase, close to the confidence limits of the determination. We have assessed the effect of these uncertainties by independently varying the three gas phase rotational constants by ± 10 % (while keeping the dipole moment fixed at 0.51 Debye), and find that the para ground state polarizability varies between 21 and 26 Å^3, while the ortho ground state polarizability varies between 28 and 38 Å^3. However the ratio of ortho and para polarizabilities is not strongly affected by changes in the rotational constants. The inelastic neutron scattering data reported in Ref. 12 display a significantly smaller uncertainty and reveal a split ortho ground state. The interaction that causes this $\sim$ 0.6 meV is currently unknown; it was therefore neglected in this work.

By taking into account higher rotational states, one calculate the dependence of the orientational polarizability on temperature. Figure S4 shows the polarizability volumes of ortho and para water versus temperature (red and blue solid curves, respectively), obtained as Boltzmann averages over the rotational ortho and para states up to J = 7. The polarizability volumes of ortho and para water separate below approximately 20 K. At high temperatures, the polarizability volumes can be approximated by a Curie law (dotted line in Fig. S4). At 50 K, the lowest temperature used to determine the dipole moment experimentally (see Fig. S1 and section IVC), the deviation between the quantum calculation and the Curie law amounts to 2.0 Å^3, while at 250 K the difference is only 0.05 Å^3.

Supplementary Methods

Synthesis and Purification

A 4:1 mixture of $H_2O@C_{60}$ and C_{60} was synthesised by "molecular surgery": a sequence of organic reactions was used to open an orifice in C_{60}, encapsulate H_2O and finally close the orifice. The $H_2O@C_{60}/C_{60}$ mixture and the C_{60} sample used in the measurements were purified by sublimation under vacuum[1].

Capacitance Cell and Measurement

Three nearly identical capacitors are obtained by fitting a 210 µm thick G10 spacer between two printed circuit boards (PCBs) with three electrodes of 13 mm diameter deposited on each board. A sample pellet is obtained by distributing 27 mg of C_{60} powder evenly across one electrode, aligning the top PCB and applying a weight of 5 tons across a 13mm diameter piston that is centered at the back of the top PCBs electrode. After pressing the first pellet, the top PCB is removed carefully, the second capacitor is filled with 27 mg of a homogenous 4:1 mixture of $H_2O@C_{60}$ and C_{60}. and the process is repeated. The thickness of the pellets obtained in this way is 300 and 260 µm for the capacitors filled with C_{60} and $H_2O@C_{60}$, respectively. The PCBs are screwed together with constant torque. Stainless steel screws and copper-berylium washers are used to minimize changes of the capacitor geometry upon cryo-cooling. The DC resistance at room temperature was larger than 20 MOhm for all capacitors. The temperature is monitored using a Cernox temperature sensor that is attached to the ground-plane at the bottom side of one of the PCBs.

The fully assembled sample cell is attached to a 3D printed nylon support using a single fixing point. The nylon support is mounted in a home-built probe that consists of a glass-reinforced plastic tube, glued into a KF50 / KF40 reducer cross (e.g. Kurt Lesker LTD) that hosts six SMA connectors and one Fischer connector for the capacitance and temperature measurements, respectively. To perform measurements below room temperature, the probe is fitted into a SpectrostatNMR cryostat (Oxford Instruments). The cryostat is equipped with a temperature controller which is connected to a computer to control and monitor the temperature.

The capacitances of the two filled capacitors are measured using the two channels of an

Analog Devices AD7746 single-chip capacitance bridge; the empty capacitor is connected to a second AD7746 capacitance bridge to provide a reference. The AD7746 operates at a fixed frequency of 16 kHz and offers a dynamic range of effectively 21 bits over 8 pF corresponding to a resolution of 4 aF or 500 ppb. The measurement is performed differentially, rendering stray capacitances to ground almost negligible. Two Arduino Uno boards are used to configure the capacitance bridges and communicate the capacitance values to an attached computer. The ortho-para conversion induces a very significant capacitance change of approximately 300 fF, so that higher resolution, although achievable[2], is not essential in studying the variations of the dielectric constant as spin conversion proceeds.

Capacitance C and dielectric constant ϵ are related by $C = \epsilon_0 \epsilon A/d$, where A and d denote the electrode area and the distance between the electrodes and ϵ_0 is the vacuum permittivity. The variation in capacitance of the empty capacitor was less than 4% over the entire temperature range from room-temperature down to 5 K. Scaling the capacitance values of the C_{60} and $H_2O@C_{60}$ capacitor values with the empty capacitor values changes the resulting ortho and para polarizability volumes by less than 1 $Å^3$.

NMR Measurement

The NMR spin conversion at 5 K was studied in a magnetic field of 14.1 T by using a cryogenic setup described previously[3]. In the reported experiment a saturation-recovery-acquire pulse sequence was used to monitor the NMR signal at regular time points. The pulse sequence consisted of a comb of 200 $\pi/2$-pulses seperated by 100 µs, followed by a recovery delay of 2 minutes and an excitation $\pi/2$ pulse. The duration of the pulses was 3 µs. The acquisition of the free induction decay started 10 µs after the excitation pulse to allow ring down of the probe. The integral of the phased spectra versus time of acquisition is shown in Fig. 3.

SUPPLEMENTARY REFERENCES

[1] Krachmalnicoff, A., Levitt, M.H. & Whitby, R.J. An optimised scalable synthesis of $H_2O@C_{60}$ and a new synthesis of $H_2@C_{60}$. *Chem. Commun.* **50**, 13037–13040 (2014).

[2] Pilla, S., Hamida, J.A. & Sullivan, N.S. Very high sensitivity ac capacitance bridge for the dielectric study of molecular solids at low temperatures. *Review of Scientific Instruments* **70**, 4055–4058 (1999).

[3] Mamone, S. *et al.* Nuclear spin conversion of water inside fullerene cages detected by low-temperature nuclear magnetic resonance. *The Journal of Chemical Physics* **140**, 194306 (2014).

[4] Bonin, K.D. & Kresin, V.V. *Electric-dipole Polarizabilities of Atoms, Molecules, and Clusters* (World Scientific Publishing Company Incorporated, 1997).

[5] Alers, G.B., Golding, B., Kortan, A.R., Haddon, R.C. & Theil, F.A. Existence of an orientational electric dipolar response in C_{60}single crystals. *Science* **257**, 511–514 (1992).

[6] Yan, F. & Wang, Y.N. The dielectric properties of polycrystalline C_{60}. *Applied Physics Letters* **72**, 3446–3448 (1998).

[7] Atkins, P. & de Paula, J. *Atkins' Physical Chemistry* (Oxford University Press, 2010).

[8] Clough, S.A., Beers, Y., Klein, G.P. & Rothman, L.S. Dipole moment of water from Stark measurements of H_2O, HDO, and D_2O. *The Journal of Chemical Physics* **59**, 2254 (1973).

[9] Ensing, B., Costanzo, F. & Silvestrelli, P.L. On the Polarity of Buckminsterfullerene with a Water Molecule Inside. *J. Phys. Chem. A* **116**, 12184–12188 (2012).

[10] Bunker, P.R. & Jensen, P. *Molecular Symmetry and Spectroscopy* (Canadian Science Publishing (NRC Research Press), 2006), 2nd revised edition.

[11] Chang, Y.P., Filsinger, F., Sartakov, B.G. & Küpper, J. CMIstark: Python package for the Stark-effect calculation and symmetry classification of linear, symmetric and asymmetric top wavefunctions in dc electric fields. *Computer Physics Communications* **185**, 339–349 (2014).

[12] Beduz, C. *et al.* Quantum rotation of ortho and para-water encapsulated in a fullerene cage. *Proceedings of the National Academy of Sciences of the United States of America* **109**, 12894–12898 (2012).

[13] Hall, R.T. & Dowling, J.M. Pure Rotational Spectrum of Water Vapor. *The Journal of Chemical Physics* **47**, 2454 (1967).
